# Spatially-resolved optical and structural properties of semi-polar $\mathrm{(11}\bar{2}\mathrm{2)}$ Al_*x*_Ga_1−*x*_N with *x* up to 0.56

**DOI:** 10.1038/s41598-017-10923-9

**Published:** 2017-09-07

**Authors:** Jochen Bruckbauer, Zhi Li, G. Naresh-Kumar, Monika Warzecha, Paul R. Edwards, Ling Jiu, Yipin Gong, Jie Bai, Tao Wang, Carol Trager-Cowan, Robert W. Martin

**Affiliations:** 1 Department of Physics, SUPA, University of Strathclyde, Glasgow, G4 0NG United Kingdom; 2 Department of Electronic and Electrical Engineering, University of Sheffield, Sheffield, S1 3JD United Kingdom; 3 Strathclyde Institute of Pharmacy and Biomedical Sciences, University of Strathclyde, Glasgow, G4 0RE United Kingdom

## Abstract

Pushing the emission wavelength of efficient ultraviolet (UV) emitters further into the deep-UV requires material with high crystal quality, while also reducing the detrimental effects of built-in electric fields. Crack-free semi-polar $\mathrm{(11}\bar{2}\mathrm{2)}$ Al_*x*_Ga_1−*x*_N epilayers with AlN contents up to *x* = 0.56 and high crystal quality were achieved using an overgrowth method employing GaN microrods on *m*-sapphire. Two dominant emission peaks were identified using cathodoluminescence hyperspectral imaging. The longer wavelength peak originates near and around chevron-shaped features, whose density is greatly increased for higher contents. The emission from the majority of the surface is dominated by the shorter wavelength peak, influenced by the presence of basal-plane stacking faults (BSFs). Due to the overgrowth technique BSFs are bunched up in parallel stripes where the lower wavelength peak is broadened and hence appears slightly redshifted compared with the higher quality regions in-between. Additionally, the density of threading dislocations in these region is one order of magnitude lower compared with areas affected by BSFs as ascertained by electron channelling contrast imaging. Overall, the luminescence properties of semi-polar AlGaN epilayers are strongly influenced by the overgrowth method, which shows that reducing the density of extended defects improves the optical performance of high AlN content AlGaN structures.

## Introduction

The versatility of III-nitride semiconductors is in part due to the extremely broad range of emission wavelengths which are made accessible by tailoring the band gap. Ultraviolet (UV) light emitters can be fabricated by alloying GaN with AlN, for applications in water purification, sterilisation, medical devices (e.g. diagnostic and treatment of skin diseases such as psoriasis), sensors and white light-emitting diodes (LEDs) through pumping of phosphors^1^. Despite significant progress in the development of deep-UV LEDs with high AlN composition, the devices suffer from low quantum efficiencies and optical output powers^2–4^. There are a number of inter-related reasons for this, including large electric fields, high concentrations of extended defects, challenges with both *n*- and *p*-type doping, poor light extraction and availability of suitable and cost-efficient substrates^5^. Traditionally, the growth of III-nitrides was carried out along the *c*-orientation or polar direction, which is heavily affected by spontaneous and piezoelectric (strain-induced) polarisations causing built-in electric fields across the active region of a quantum well (QW) structure^6^. These electric fields are the cause of the quantum-confined Stark effect (QCSE), which reduces the quantum efficiency, and its effect increases with increasing AlN fraction. Growth of Al_*x*_Ga_1−*x*_N in non- or semi-polar orientations is of interest for removing or decreasing the effect of the QCSE, as already successfully employed for visible devices using the InGaN alloy system^7–9^. However, moving away from the polar orientation leads to a high density of extended defects such as stacking faults (SFs) and misfit dislocations in addition to threading dislocations (TDs) also present in *c*-orientated hetero-epitaxial nitride materials^10–12^. Another issue is the lack of cost-efficient AlN substrates and the use of non-native, lattice mismatched-substrates (e.g. sapphire or SiC) for high AlN content AlGaN results in high dislocation densities and cracking due to tensile strain^13^. Besides using expensive AlN substrates, the defect density can be lowered by using patterned substrates, by employing an overgrowth method to block defects, which at the same time can reduce the strain and prevent cracking, or by fabricating nanostructures^14–16^. It is important to note that the above mentioned effects become more severe for deep-UV emitters with even higher AlN concentration.

In order to combat these effects, semi-polar $\mathrm{(11}\bar{2}\mathrm{2)}$ AlGaN epilayers have been grown on semi-polar $\mathrm{(11}\bar{2}\mathrm{2)}$ GaN overgrown on GaN microrod arrays^17, 18^. Although the growth along the semi-/non-polar directions presents a promising solution, producing material with good crystal quality proved a challenge. Over the last decade a considerable amount of effort was spent on strain management, identifying and reducing the number of extended defects by using different growth methods and multilayer structures, which has led to semi-polar AlGaN with greatly improved crystal quality^12, 17–21^.

Most reports in the literature on semi-polar AlGaN rely on luminescence information from area-averaged measurements, such as photo- and electroluminescence or absorption measurements^4, 18, 19, 21^. Structural data on extended defects is generally obtained by time-intensive transmission electron microscopy (TEM), which is a destructive technique due to sample preparation. In this work, the optical and structural properties of crack-free semi-polar $\mathrm{(11}\bar{2}\mathrm{2)}$ Al_*x*_Ga_1−*x*_N epilayers grown on GaN microrod templates with high AlN contents ranging from *x* = 0.38 to 0.56 and significantly improved crystal quality were investigated. Characterising semi-polar AlGaN epilayers with high AlN content is a precursor to understanding the effects of AlN incorporation on the optical properties of semi-polar quantum wells in deep-UV LEDs. Details of the sample structure can be found in the experimental methods at the end of the manuscript. Combining cathodoluminescence (CL) hyperspectral imaging and electron channelling contrast imaging (ECCI) in a scanning electron microscope (SEM) allowed us to correlate defects present in the material with their spatially- and spectrally-resolved luminescence characteristics without sample preparation^22^.

## Results and Discussion

**Figure 1:**
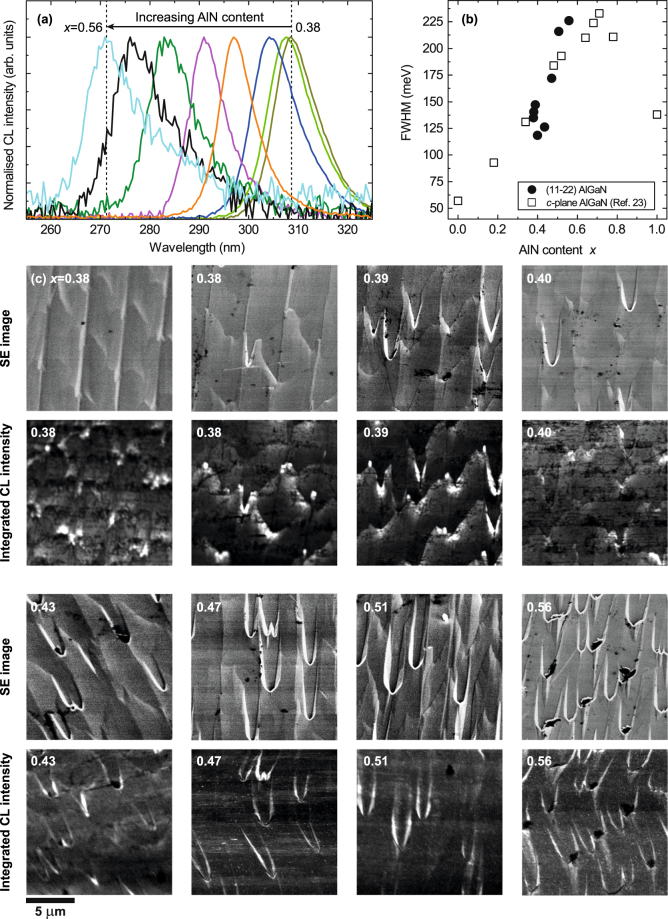
(**a**) Normalised room temperature CL spectra of the semi-polar $\mathrm{(11}\bar{2}\mathrm{2)}$ Al_*x*_Ga_1−*x*_N epilayers with AlN concentrations raging from *x* = 0.38 to 0.56. (**b**) FWHM as a function of composition of the $\mathrm{(11}\bar{2}\mathrm{2)}$ AlGaN and *c*-plane AlGaN from Ref. 23. (**c**) SE and integrated CL intensity images of the AlGaN peaks. The CL was recorded more slowly than the SE images, which were acquired first from the same areas, causing distortion due to sample drift in the samples with higher AlN fraction.

For an initial evaluation, room temperature CL measurements were performed over a relatively large area of side length 15 *μ*m on all AlGaN samples. From the CL data sets area-averaged mean spectra were calculated, which are shown in Fig. 1(a) on a normalised intensity scale. Increasing the AlN composition shifts the near band edge (NBE) emission from about 309 nm for the sample with the lowest concentration (*x* = 0.38) to 271 nm for the AlGaN layer with *x* = 0.56. Increasing the AlN content also leads to a decrease in emission intensity, an effect which is most pronounced for the three samples with the highest AlN contents as seen in Fig. 1(a) by the strongly reduced signal-to-noise ratio. The trend of the full width at half maximum (FWHM) taken from the mean spectra is shown in Fig. 1(b) in comparison with room temperature photoluminescence data from *c*-plane AlGaN. Increasing the composition leads to an increase in linewidth starting from either binary compound. This is caused by compositional fluctuations due to the degradation of the crystal and non-uniform incorporation of aluminium atoms as the concentration is increased. It is interesting to note that the semi-polar AlGaN samples exhibit comparable linewidths to their *c*-plane counterparts as shown in Fig. 1(b) ^23^.

Secondary electron (SE) images were recorded from the same areas from which the CL was recorded and are displayed in Fig. 1(c) in order of increasing AlN content. The surface morphology is strongly influenced by a change in composition. Chevrons or arrowhead features are observed on the surface of all AlGaN samples. Chevrons are three-dimensional objects with facets of $\{10\bar{1}1\}$-orientation, which is the same orientation as the facets of V-defects in *c*-orientated QWs^24^. The density of chevrons increases dramatically for higher AlN contents. The sample with the largest content of *x* = 0.56 even exhibits pits at the tips of some of the chevrons. Chevrons have already been reported for semi-polar nitride structures, which have been grown using an overgrowth technique^15, 24–28^. These chevrons are already present in the semi-polar GaN templates which were used for the growth of the AlGaN^29^. Chevrons form due to imperfect coalescence during the overgrowth stage when two growth fronts with different growth rates meet^28^. Growth on the *c*-plane is faster compared with the *a*-plane and it eventually overgrows the $\mathrm{(11}\bar{2}\mathrm{2)}$ surface leading to steps on the surface. The influence of the different growth rates on the structure overgrowing the microrods can be seen in Fig. 1(b) in Ref. 30. The figure also shows that at the beginning of the coalescence the different facets of the overgrown structure initially meet in a V-shape leading to chevrons on the surface. Chevrons have a strong impact on the optical properties. CL is a useful tool to spatially resolve their luminescence and investigate the incorporation of AlN around their different facets as discussed in the following paragraphs^31^. Chevrons are also observed in semi-polar InGaN/GaN QW structures and LEDs emitting in the visible region and CL imaging showed the direct influence on the light emission^32, 33^. As shown later, the tips of the chevrons are aligned along the $\mathrm{[11}\bar{2}\mathrm{3]}$-direction.

Integrated CL intensity images of the entire AlGaN emission peak were calculated from the hyperspectral data set in order to estimate the total emission uniformity across the samples and are shown in Fig. 1(c). Several different features can be identified in the CL intensity images. First of all, the emission is strongly influenced by the presence of the chevrons, with the regions corresponding to the chevrons exhibiting much stronger luminescence compared with the surrounding area. Secondly, darker and brighter stripes perpendicular to the direction of the chevrons can be observed for the samples with an AlN concentration up to about 40%. Additionally, dark spots associated with non-radiative recombination at TDs can be observed^22^.

**Figure 2:**
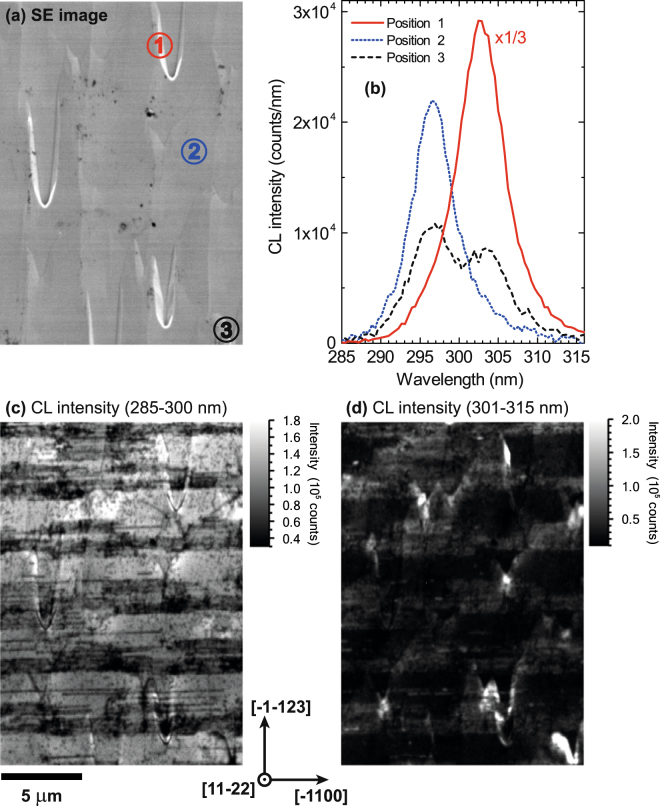
CL data from the semi-polar Al*x*Ga_1−*x*_N epilayer with *x* = 0.40: (**a**) SE image. (**b**) Example CL spectra from positions 1–3 marked in the SE image. The spectrum from position 1 is scaled down by a factor of 3 compared with the other two spectra. Integrated CL intensity images for the wavelength ranges (**c**) 285–300 nm and (**d**) 301–315 nm. Reduced luminescence in horizontal stripes corresponding to BSF affected areas are clearly visible in (**c**). The crystallographic directions are marked according to the direction of the BSFs by comparison with TEM images in Ref. 30.

On closer inspection of the individual CL spectra, a range of emission peaks can be observed originating from different positions on the sample surface. In the SE image in Fig. 2(a) three positions are marked from which individual CL spectra were extracted from the hyperspectral data set from the Al_0.40_Ga_0.60_N sample as shown in Fig. 2(b). Position 1 shows one arm of a chevron, position 2 is representative of the majority of the surface and position 3 is near a chevron. Due to the intense emission from the arm of a chevron (position 1) the spectrum was scaled by a factor of 1/3 compared with the other two spectra. From the spectra in Fig. 2(b) two dominant emission peaks can be identified. The longer wavelength peak, centred around 303 nm, is stronger near and around chevrons (positions 1 and 3), whereas the weaker and shorter wavelength peak at approximately 297 nm is emitted from the majority of the sample surface (position 2). Due to the proximity and overlap of the two emission peaks, peak fitting did not produce informative luminescence maps. Therefore, integrated CL intensity images for the wavelength ranges of 285–300 nm and 301–315 nm were generated from the full data set and displayed in Fig. 2(c) and (d), respectively. Compared with the CL intensity images in Fig. 1(c) the different features appearing in the two CL images appear much clearer now and can be attributed to the different emission peaks. The longer wavelength emission is only observed on and near chevrons. Non-radiative recombination at extended defects (i.e. dark lines and spots) is more visible in Fig. 2(c), because the majority of the area is dominated by the emission with the shorter wavelength and the contributions of the longer wavelength peak to the image have been removed.

TEM measurements of the semi-polar $\mathrm{(11}\bar{2}\mathrm{2)}$ GaN template overgrown on microrod arrays showed striped regions with and without the presence of basal-plane stacking faults (BSFs)^30^. These stripes are parallel to the $[\bar{1}\mathrm{100]}$- or *m*-direction and perpendicular to the $[\bar{1}\bar{1}\mathrm{23]}$-direction. Due to the orientation of the (Al)GaN film the BSFs are tilted at an angle of 58.4° from the $\mathrm{(11}\bar{2}\mathrm{2)}$ surface plane. The $[\bar{1}\mathrm{100]}$- and $[\bar{1}\bar{1}\mathrm{23]}$-direction are perpendicular to each other and within the $\mathrm{(11}\bar{2}\mathrm{2)}$ surface plane. Due to the overgrowth technique the BSFs are localised to stripes separated by areas largely free of BSFs. Due to a second coalescence stage the BSF density periodically changes along a stripe. As shown in Fig. 2(c) BSFs are centres for non-radiative recombination and appear as thinner dark lines bunched up in wider stripes in the CL intensity image. Since the luminescence at chevrons is redshifted, they appear dark in the CL image in Fig. 2(c), which shows the intensity at shorter wavelengths only.

**Figure 3:**
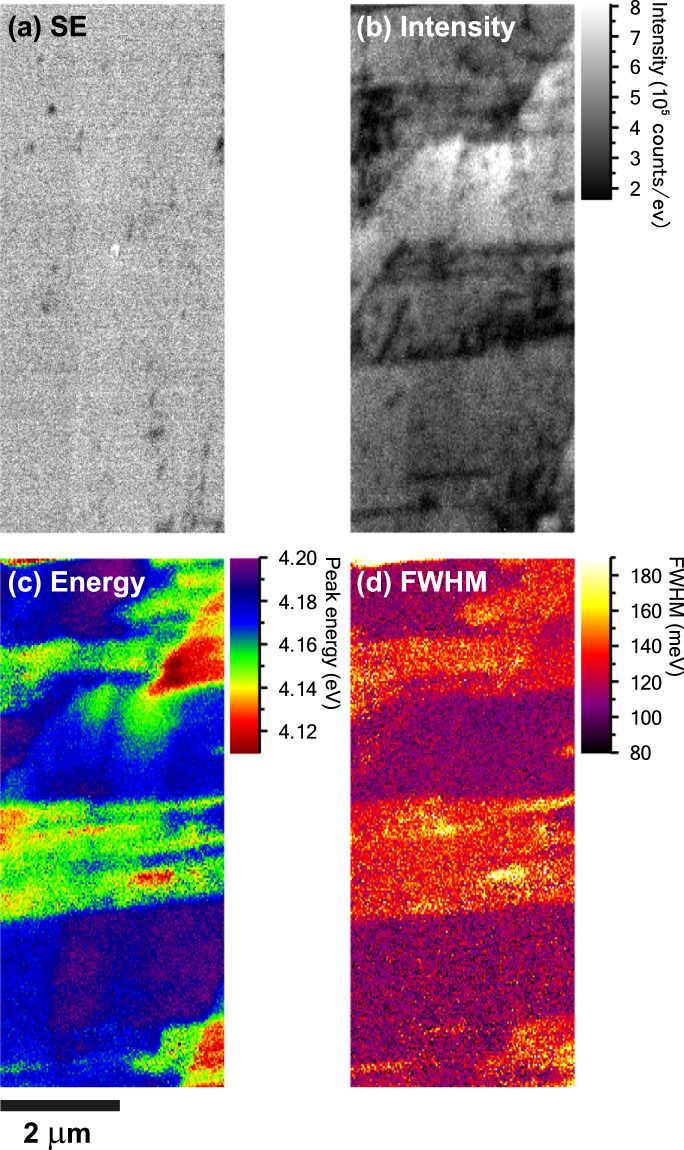
High resolution CL imaging of the Al_*x*_Ga_1−*x*_N sample with *x* = 0.40: (**a**) SE image and CL images through peak fitting: (**b**) peak intensity, (**c**) peak energy and (**d**) FWHM. (**a**,**c** and **d**) from Ref. 17. (Creative Commons Attribution (CC BY) license: http://creativecommons.org/licenses/by/4.0/).

In order to investigate the influence of the BSFs on the luminescence properties of the semi-polar AlGaN, higher resolution CL measurements were carried out between chevrons. Since there is only one emission peak present in this region, the entire data set was fitted using a Gaussian function and least-squares fitting method. The resultant fitting parameters of the peak height (intensity), peak position (energy) and FWHM are shown in Fig. 3 together with the SE image of the same area. BSFs and TDs are better resolved in the higher resolution CL images gained through peak fitting compared with the CL intensity image in Fig. 2(c). Comparing the dark regions in the CL intensity map in Fig. 3(b) where BSFs are present to the peak energy and FWHM image in Fig. 3(c) and (d), respectively, there is a clear correlation. The peak is slightly redshifted in the BSF affected area from about 296 nm (4.19 eV) to 299 nm (4.15 eV) and the peak width increases from approximately 109 meV to 137 meV. On closer inspection of the individual CL spectra from these two regions it can the seen that the energy shift is not real, but rather attributed to a peak broadening on the lower energy side of the emission peak. Although BSF-related luminescence on the lower energy side is quenched at room temperature, the presence of BSFs leads to stronger exciton localisation compared with the NBE emission^18^. The crystal quality in the BSF-affected area is most likely lower compared with the neighbouring areas, which causes a slight broadening of the emission peak and the apparent redshift visible in the CL energy image in Fig. 3(c).

**Figure 4:**
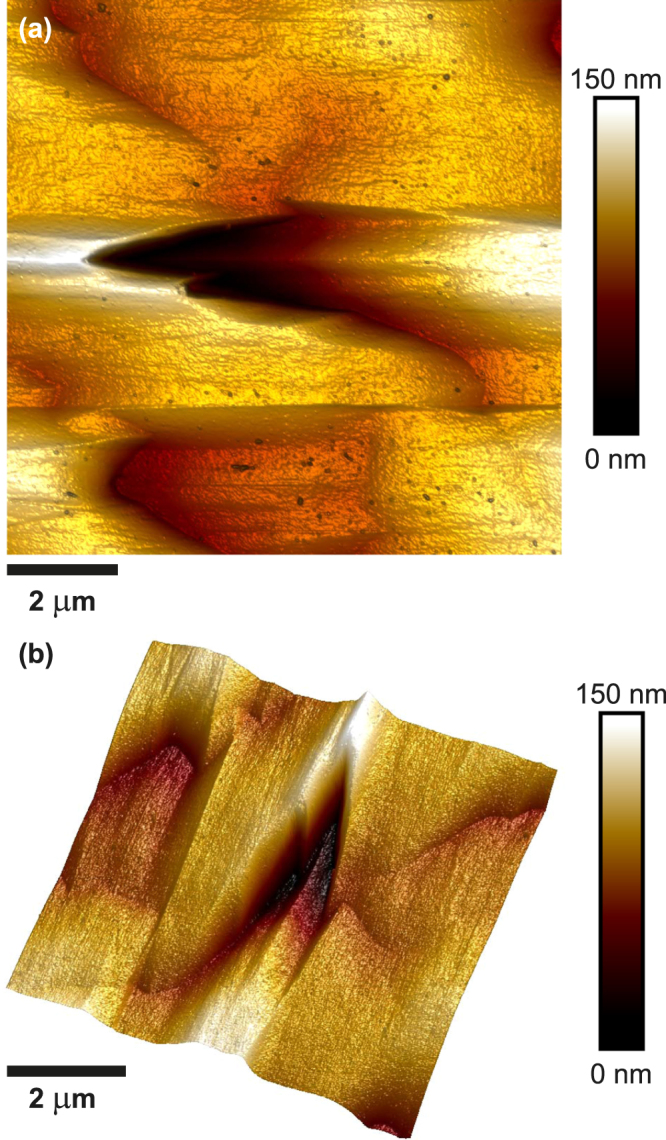
Atomic force microscope image of a chevron occurring in a semi-polar Al_*x*_Ga_1−*x*_N sample with *x* = 0.40: (**a**) top-down view and (**b**) three-dimensional representation.

The emission intensity at chevrons is much stronger and redshifted by about 6 nm compared with the surrounding regions. The longer wavelength peak is only present on and near chevrons as shown by the CL intensity image centred around the 303 nm emission in Fig. 2(d). Comparing the orientation of the chevrons to the dark stripes from the BSF affected region it can be inferred that the tips of the chevrons are aligned along the $\mathrm{[11}\bar{2}\mathrm{3]}$-direction. For V-defects it has been shown that the emission changes due to a change of the growth conditions on the facets^34^. In a similar way the growth conditions, such as the growth rate, have changed on the facets of the chevrons leading to a different incorporation of Al atoms and therefore a different AlN content. Given that the emission peak appears at a longer wavelength compared with the surrounding area, it is inferred that the AlN composition and hence the band gap is lower on the facets of the chevrons than the rest of the sample. The reduced AlN content on the facets of the chevrons causes a local improvement in crystal quality, contributing to the increase in emission intensity in those regions. Additionally, the chevrons will act as a sink for carriers due to the locally smaller band gap on the facets. Wavelength- and energy-dispersive X-ray spectroscopy (WDX/EDX) measurements were carried out on regions displaying chevrons in order to investigate the possible change in AlN content. The results, however, were inconclusive, which is partly due to the strong topography in this region. A change in the surface orientation leads to different take-off angles for the X-rays, with lower angles causing increased absorption of X-rays. Atomic force microscope (AFM) measurements of the AlGaN sample with 40% AlN were conducted and an AFM image of a chevron is displayed in Fig. 4. The approximate angle between the two arms of the chevron is 25°. The area around a chevron is quite undulating and depending on where a measurement is taken, the depth of a chevron is about 60–100 nm.

**Figure 5:**
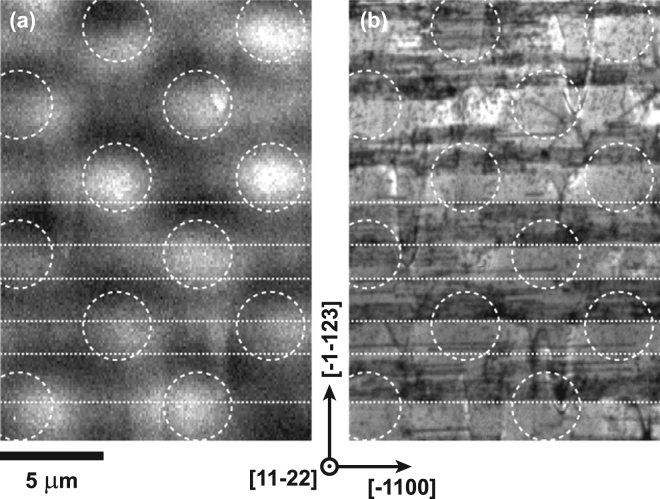
Integrated CL intensity image (**a**) of the GaN NBE emission peak (355–375 nm) taken at 30 kV and (**b**) of the shorter AlGaN emission band (285–300) taken at 5 kV from the semi-polar Al_*x*_Ga_1−*x*_N sample with *x* = 0.40. In order to minimise the influence of surface contamination the CL data set at 5 kV was recorded before the one at 30 kV from the same area. The circles roughly mark the underlying GaN microrod template and the dotted lines regions with and without BSFs.

The microrod template and overgrowth mechanism serve to reduce the density of extended defects, but are also the cause of the observed luminescence variations (i.e. stripes) as shown in the CL images. In order to probe the luminescence behaviour of the GaN template an acceleration voltage of 30 kV was chosen for the electron beam to penetrate the 2.7 *μ*m thick AlGaN layer. At this beam voltage the spatial resolution is drastically reduced due to the excitation volume compared with the previous 5 kV measurements. CL data sets at 5 kV and 30 kV were recorded from the same area in order to compare the luminescence characteristics of the AlGaN layer and its GaN template. The 5 kV measurement was performed first in order to minimise the influence of surface contamination. The CL integrated intensity images of the GaN NBE emission peak (355–375 nm) and the shorter wavelength AlGaN emission peak (285–300 nm) are shown in Fig. 5(a) and (b), respectively. The GaN intensity image shows periodic intensity variations that can be related to the 4 *μ*m diameter microrod array, which is superimposed on both CL images as dashed circles. The position of the dark stripes with respect to the microrod array depends on the film thickness since the BSFs intersect the surface at an angle of 58.4°. The change in BSF density along a stripe, as mentioned before, might also be reflected by a change in intensity of the GaN NBE emission along a dark strip (marked by the dotted lines).

**Figure 6:**
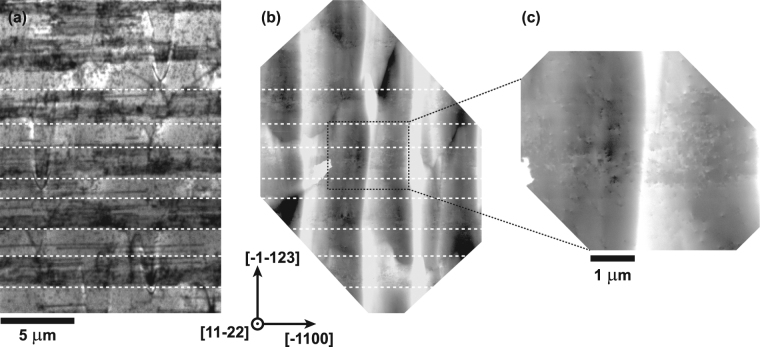
(**a**) Integrated CL intensity image of the shorter wavelength emission (285–300 nm) and (**b**) Electron channelling contrast image on the same scale but a different area of the same semi-polar Al_*x*_Ga_1−*x*_N sample with *x* = 0.40. The dashed lines indicate regions with and without BSFs. (**c**) Higher resolution ECC image of the area marked in (**b**) showing either individual TDs or partial dislocation terminating at the end of a BSF.

Structural defects were investigated by performing ECCI on the Al_*x*_Ga_1−*x*_N sample with *x* = 0.40. The resultant electron channelling contrast images were then compared with the CL images from the same sample. Figure 6(a) and (b) show the CL intensity image of the shorter wavelength emission (285–300 nm) and the ECC image of a similar (but not the same) area of the same sample, respectively. To better resolve dislocations, a higher resolution ECC image was recorded as shown in Fig. 6(c). Similar to the CL image in Fig. 6(a) the ECC image in Fig. 6(b) exhibits bands of extended defects, which are highlighted with dashed lines. The bright white elongated features perpendicular to these bands of extended defects are the topographic contrast due to large range undulations on the surface of the sample, which can also be observed in the AFM image in Fig. 4. The high resolution ECC image in Fig. 6(c) of the region associated with BSFs exhibits a large number a spots with black–white contrast. These spots are either individual TDs or partial dislocations terminating at the end of a BSF. Resolving BSFs in this sample was not possible, because the contrast from the large range surface undulations made it impossible to visualise BSFs, which run parallel to the surface. ECCI of the semi-polar GaN template (i.e. a similar structure without the thick AlGaN layer), which exhibits a much smoother surface morphology, showed that BSFs are present in the regions which show dark stripes in CL. This is also confirmed by plan-view TEM^29, 30^. From several ECC images like the one in Fig. 6(c) an average TD density of approximately 2 × 10^9^ cm^−2^ and 2 × 10^8^ cm^−2^ was estimated for areas with (dark stripe) and without BSFs (brighter stripe), respectively. This difference in density might be due to double counting of partial dislocations terminating BSFs. Combining CL and ECCI confirmed that the dark stripes composed of many thinner dark lines in CL are regions where BSFs are present. These regions also showed an increased density of TDs compared with the BSF-free regions, which appear as brighter stripes in CL intensity images.

## Summary

In summary, the luminescence characteristics of semi-polar $\mathrm{(11}\bar{2}\mathrm{2)}$ AlGaN samples with AlN contents of *x* = 0.38–0.56 were investigated using CL hyperspectral imaging and were correlated to structural information on extended defects gained by ECCI. The crystal quality of the AlGaN was significantly improved by using an overgrowth method employing GaN microrod templates on *m*-sapphire in order to reduce the BSF density. All samples exhibited chevrons with their density increasing for higher AlN fractions. CL imaging revealed two dominant emission peaks. The longer wavelength peak is only present near and around chevrons and the shorter wavelength peak is emitted from the majority of the sample. Chevrons are three-dimensional features of semi-polar materials that form during the overgrowth stage. From the redshifted emission peak it can be inferred that the AlN content on the facets of the chevrons is lower compared with the rest of the sample due to locally different growth conditions. The shorter wavelength emission peak is strongly influenced by the microrod template. Due to the overgrowth BSFs are present as periodic stripes, which appear as dark stripes in CL intensity images of the shorter wavelength peak since they act as centres for non-radiative recombination. Within these stripes the emission intensity is reduced and broadened leading to an apparent redshift in this area of reduced crystal quality. Additionally, dark spots associated with non-radiative recombination at TDs are present across the entire sample. ECCI determined that the density of TDs is one order of magnitude lower in BSF-free regions (brighter stripes) compared with the regions associated with BSFs (darker stripes). Using the overgrowth techniques significantly improves crystal quality by reducing the BSF density and hence enhances the optical performance, which allows the realisation of deep-UV emitters for a wide range of applications from sensing to medical instruments.

## Experimental Methods

The samples were grown by metalorganic chemical vapour deposition using a two-step overgrowth method. A series of semi-polar AlGaN layers with different AlN concentrations were deposited on GaN overgrown on microrod templates. For the GaN microrod template a high temperature AlN buffer was grown on *m*-plane sapphire substrates followed by a 400 nm thick $\mathrm{(11}\bar{2}\mathrm{2)}$ GaN layer^35^, which was processed into regularly-arrayed microrods of 4 *μ*m diameter and pitch using a standard photolithography technique and dry-etching processes^8, 36^. The microrod array was overgrown with semi-polar GaN until the GaN layer was nearly-but not fully-coalesced at a thickness of approximately 2.1 *μ*m at which point the growth of about 2.7 *μ*m thick semi-polar $\mathrm{(11}\bar{2}\mathrm{2)}$ AlGaN was initiated. The AlN concentration ($x=0.38\mbox{--}0.56$) was controlled by varying the flow-rates of trimethylaluminium and trimethylgallium. The AlN concentration in the AlGaN layer was estimated from the position of the AlGaN peak with respect to the angle of sapphire or GaN in a $\omega \mathrm{/2}\theta$-scan using X-ray diffraction (XRD). Further details on the growth can be found elsewhere^18^.

The custom-built CL hyperspectral imaging system is attached to a variable pressure field emission SEM (FEI Quanta 250). The sample is placed at an angle of 45° with respected to the incident electron beam. The emitted light is collected by a Cassegrain reflecting objective with its optical axis perpendicular to the direction of the electron beam, dispersed with a 1/8 m focal length spectrometer (Oriel MS125) and collected using a 1600-channel electron multiplying charge-coupled device (Andor Newton). The wavelength calibration is performed using two lines in the spectrum of a mercury lamp. The wavelength uncertainty is less than 0.5 nm and the resolution is approximately 2 nm. CL is carried out at room temperature in hyperspectral imaging mode, meaning that an entire spectrally-resolved luminescence spectrum is collected per pixel with a spatial resolution approaching 10 nm^31, 34, 37^. From the hyperspectral data set, two-dimensional CL images are extracted by plotting the integrated intensity of a certain wavelength range and by least-squares peak fitting for images of the peak intensity, energy and linewidth. The CL was acquired at room temperature at 5 kV unless otherwise stated.

ECCI is a non-destructive, diffraction technique performed in the SEM^38^. ECC images are generally constructed by measuring the intensity of the backscattered electrons (BSEs) as the electron beam scans across the surface of a suitably-orientated sample. Any changes in crystallographic orientation and local strain can be monitored by the variation in the BSE intensity causing a change in contrast in an ECC image. This allows the imaging of low-angle tilt and rotation boundaries, atomic steps and extended defects (e.g. TDs). ECCI is carried out in a forward scattering geometry in a field emission SEM (FEI Sirion 200), equipped with an electron-sensitive diode and a custom-built signal amplifier. ECC images were acquired with a beam current of about 2.5 nA, a beam divergence of about 4 mrad and an electron beam energy of 30 keV.

Characterisation of the surface morphology was performed using atomic force microscopy (AFM, Bruker Dimension with Icon scanner) in PeakForce tapping mode with ScanAsyst Air probes (Bruker, nominal tip radius of 2 nm and nominal spring constant of *k* = 0.4 N/m).

### Data availability

The data associated with this research are available at http://dx.doi.org/10.15129/5f70bdd2-75aa-4cbd-a581-c3cbd8b5a223 or from the corresponding author.
